# Effectiveness of Combined General Rehabilitation Gymnastics and Muscle Energy Techniques in Older Women with Chronic Low Back Pain

**DOI:** 10.1155/2019/2060987

**Published:** 2019-01-23

**Authors:** Michał Wendt, Krystyna Cieślik, Jacek Lewandowski, Małgorzata Waszak

**Affiliations:** ^1^Department of Functional Anatomy, Poznan University of Physical Education, Poznan/61-871, Poland; ^2^Department of Musculoskeletal Rehabilitation, Poznan University of Physical Education, Poznan/61-871, Poland

## Abstract

**Objective:**

The aim of this study was to determine the effect of general rehabilitation gymnastics on subjective and objective characteristics of locomotor system in older women with chronic LBP. To satisfy this goal, the outcomes in exercising women were compared with the results of nonexercising controls.

**Material and Methods:**

The study group included 21 women with chronic LBP (age 65-75 years), participating in a 3-year general rehabilitation program combining strength, stretching, endurance, balance, and stabilization exercises with Muscle Energy Techniques (MET). Control group included 20 women with chronic LBP, who neither undertook the gymnastics nor participated in other forms of physical activity. The list of outcome measures included pain severity (Numeric Rating Scale), limitations in the activities of daily living (Oswestry Disability Index and Roland-Morris Disability Questionnaire), mobility of all spinal segments (tensometric electrogoniometry), and bioelectrical activity of back muscles (kinesiologic electromyography).

**Results:**

Exercising women presented with lesser severity of current pain (by 62%, p<0.001) and pain experienced during the last three months (by 32.5%, p<0.001), reported less ailments during the last three months, and had fewer limitations in the activities of daily living (a 30% decrease in Oswestry Disability Index, p<0.05, and a 65% decrease in Roland-Morris Disability Questionnaire scores, p<0.001) than the controls. Moreover, they showed significantly higher values of nearly all spondylometric parameters except for cervical lateral flexion. The study groups did not differ in the amplitudes of bioelectrical signal from the back muscles.

**Conclusions:**

These findings may point to beneficial effects of the combined exercise program.

## 1. Introduction

The most common musculoskeletal disorder found in this age group is low back pain (LBP) [1]. LBP is the third most prevalent chronic disease among the elderly [2]. It occurs in 36-70% of older persons with musculoskeletal ailments [3, 4].

LBP is defined as pain in the back from the level of the lowest rib down to the gluteal fold [5]. The pain may be unilateral, bilateral, or central or vary in location. In chronic form, LBP persists for at least three months [5]. LBP leads to impairment of spinal mobility and limitations in patient's functioning [5]. According to literature, a primary source of pain in LBP is pathological changes of intervertebral discs [6, 7]. A key role in LBP pathogenesis is played by inadequate hydration of nucleus pulposus and structural changes within annulus fibrosus [8]. This broadly understood pain syndrome is sometimes referred to as a multidimensional illness, as it may affect patient functioning not only in physical, but also in psychological and social spheres. LBP may be a cause of anxiety and depression and impose limitations in one's social and professional activities [9].

Published evidence suggests that there is a link between muscle weakness, low physical activity, and increased risk of back pain [10, 11]. Many previous studies compared the effectiveness of various training systems in the management of chronic LBP in persons of various age. However, such research has rarely been conducted among the older subjects. Only few published studies analyzed the effectiveness of combined training programs in this age group [12]. To the best of our knowledge, the effects of combined strength, stretching, endurance, balance, and stabilization exercises with the elements of Muscle Energy Techniques (MET) have not been studied in older persons with chronic LBP thus far.

The aim of this study was to analyze the effects of general rehabilitation gymnastics program with various types of exercises on the subjective and objective indices of locomotor system status in older women with chronic LBP. To satisfy this goal, we compared the outcomes in women who participated in the general rehabilitation gymnastics program with the results of nonexercising controls.

## 2. Material and Methods

The study included 21 women between 65 and 75 years of age, who participated in a 3-year biweekly general rehabilitation gymnastics program at “Antidotum” NFZ Rehabilitation Center. Aside from the gymnastics, the study subjects did not undertake any other forms of physical activity. Control group was comprised of 20 women between 65 and 75 years of age, who did not practice general rehabilitation gymnastics and other forms of sport or recreational physical activity. The selection of women participating in the study was conditioned by meeting specific conditions. The research group consisted of 21 out of 38 women, who during the period of 3 years have regularly participated general rehabilitation gymnastics program with the elements of Muscle Energy Techniques (MET). The program was conducted by a physiotherapist specializing in this method. The control group consisted of women who were enrolled to “Antidotum” NFZ Rehabilitation Center (N=86). They were chosen before starting rehabilitation based on their responses to the initial medical interview.

### 2.1. Inclusion and Exclusion Criteria

**Figure 1:**
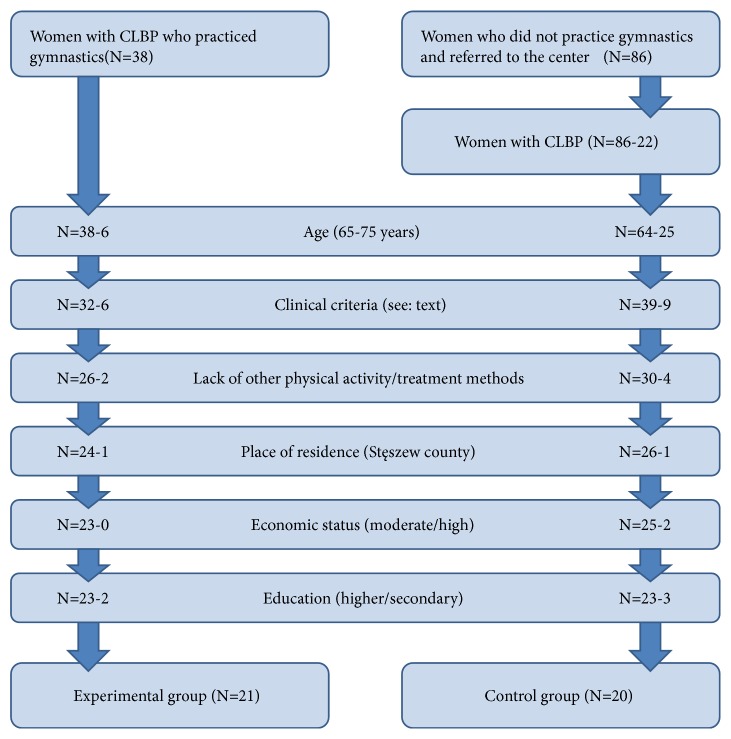
Diagram illustrating participant flow to the study groups (exercising group and control group).

Selection of women to both study groups was based on a detailed and thorough analysis of clinical and demographic criteria. The list of demographic criteria included age, place of residence, economic status and education. Both groups included solely women aged between 65 and 75 years of age, inhabitants of Stęszew county (a total of 15 000 inhabitants), with moderate or high economic status and secondary or higher education. Detailed information about the participant flow to both study groups is presented in Figure 1.

A clinical inclusion criterion to the study groups was presence of a chronic lower back pain (CLBP) persisting for more than three months. Lower back pain was defined as a unilateral or bilateral pain the intensity of which changed depending on body position and time of the day), located between the level of the lowest (XII) rib down and the gluteal fold. Also women with concomitant pain in one or both buttocks were eligible for the study. Presence of a concomitant pain/numbness/muscle weakness in one or both lower extremities, suggestive of disc-root conflict, was a considered a clinical exclusion criterion. Another exclusion criterion was occurrence of a persistent pain the intensity of which did not change depending on body position and time of the day. Also women with concomitant pain in cervical and/or thoracic spine were not qualified to the study. Finally, a present or past history of gross pathologies, e.g., osteoporosis, spinal infections, neoplasms, cauda equina syndrome, spinal fractures, or surgeries, disqualified from participation in the study.

Detailed characteristics of the study participants are presented in Table 1.

### 2.2. General Rehabilitation Gymnastic Sessions

General rehabilitation gymnastics sessions took place twice a week and lasted 45 min each. The intensity of the exercise was moderate. The program included strength, endurance, stretching, balance and stabilization exercises. The subjects performed bodyweight resistance exercises and exercises with Thera-Band resistance bands (red bands, i.e., with low resistance). The aim of the resistance training was to strengthen some specific muscle groups, such as erector spinae muscles, rectus abdominis muscle, abdominal internal and external oblique muscles, transverse abdominal muscle, deltoid muscle, triceps brachii muscle, biceps brachii muscle, rhomboid muscle, serratus anterior muscle, infraspinatus muscle, teres minor muscle, quadriceps femoris muscle, gluteal muscles (gluteus maximus, gluteus medius, and gluteus minimus), tibialis anterior muscle, and flexor digitorum longus muscle. During the resistance training, subjects performed isometric, concentric, and eccentric muscle work. The stretching exercises involved primarily trapezius muscle (descending part), scalene muscles (anterior, middle, and posterior scalene), sternocleidomastoid muscle, pectoralis major muscle, latissimus dorsi muscle, teres major muscle, suboccipital muscle, spinal rotators and erector spinae muscles (especially thoracic part), ischiocrural muscles (hamstring), rectus abdominis muscle, piriformis muscle, and triceps surae muscle. The stretching exercises included the elements of MET, such as Contact Relax (CR) and Contract-Relax-Contract (CRC) techniques based on neurophysiological mechanisms of postisometric relaxation and reciprocal inhibition [13]. Each standard session of general rehabilitation gymnastics included a 10-min warm-up to prepare the muscles for proper training work. The exercises started in various positions, e.g., standing, kneeling, and lying down (prone, supine, and lateral recumbent position). The last 5 min of each session was spent on cooling and relaxation exercises performed lying down, and breathing exercises. The program of the training was adjusted to individual condition and performance level of each participant. The training groups consisted of 10-12 subjects, which enabled the supervisor to control correctness of each exercises. The supervisors were adequately qualified persons, with master's degree in physiotherapy.

### 2.3. Measurement Methods

Selected characteristics of women from the exercising and nonexercising group were determined with objective and subjective methods. Both subjective and objective methods were conducted by a blinded assessor.

#### 2.3.1. Objective Methods

*Tensometric Electrogoniometry. *Spinal mobility was determined with Penny & Giles tensometric electrogoniometer in Boocock modification [14]. The measurements were taken using the methodology described by Lewandowski [15], with two tensometric sensors: SG-series biaxial sensor and Q-series single axis sensor. The examination started with the patient standing upright, with body weight distributed evenly between both feet, arms along the trunk, and head in horizontal plane. The sensors were attached to subject's skin with double-sided medical tape. To determine spinal mobility in various segments, the sensors were places along the long axis of the body, at the spinous processes. During the measurement of cervical spine mobility, lower edge of the upper sensor was placed on the external occipital protuberance and the upper edge of the lower sensor on top of the spinous process C7. During the measurement of thoracic spine mobility, lower edge of the upper sensor and upper edge of the lower sensor were placed on tops of spinous processes Th1 and Th12, respectively. During the measurement of lumbar spine mobility, lower edge of the upper sensor was placed on top of the spinous process Th12 and the upper edge of the lower sensor at the base of the sacral bone (S1) [15]. The mobility of each spinal segment was examined during flexion, extension, right and left side-bending, and right and left rotation.

*Surface Electromyography (EMG). *Kinesiologic electromyography was carried out with an 8-channel electromyographic system with plate electrodes (model W4X8, Biometrics Ltd). The results were recorded using DataLog Bluetooth V7.5 software (Biometrics Ltd). During the examination, two multiple-use surface electrodes (type SX230 1000) were attached with an adhesive tape, after removal of body hair from a 2 cm x 1 cm area of intact skin, disinfection of electrode surface, and wiping the skin a few times with salicylic alcohol to reduce its resistance. The reference electrode (type R230, Biometrics Ltd) was fixed at the distal end of the radius (Lister's tubercle region) with an elastic band. The examination involved lumbar segment of the longissimus muscle, both right- and left-sided bundles. The electrodes were placed according to the international guidelines published by SENIAM [16]. The examination lasted 10 min, was painless and noninvasive and did not require subject's exposure to any additional electric stimulation. The measurement was preceded by a 10-min warm-up of the key muscle groups. The examination took place in the morning, in a separate room.

The list of determined electromyographic parameters included the amplitude of bioelectrical signal from the longissimus muscle, expressed in microvolt. Avoiding a confounding effect associated with measurement conditions, the results were normalized to a reference Maximum Voluntary Contraction (MVC) and expressed in percent [17]. After fixation of the electrodes and preparation of the system, MVC of the longissimus was determined according to the international SENIAM guidelines [16]. The examination started with the subject lying down in a prone position, with arms crossed under the chin, and extended legs. The EMG recordings were obtained during three active extensions of the spine, each lasting 3 seconds, with 30-second intervals in between. MVC, corresponding to 100% neuromuscular activation of the longissimus, was calculated as the mean amplitude for the three repetitions, either for the whole muscle or for its right- and left-sided bundles.

Then, the subject was examined during standing up from a chair, sitting down on a chair, lifting up and putting down a 2 kg rehabilitation roller. Each test was conducted at an individually-adjusted pace, and included three repetitions with 30-second intervals in between. The test started on the examiner's command. Lifting up and putting down the rehabilitation roller, the subjects kept their back straight and flexed their hips. The result of each test was presented as the mean amplitude for the three repetitions, either for the whole muscle or for its right- and left-sided bundles. The results were normalized to MVC, to estimate the degree of the muscle involvement during various activities (standing up, sitting down, lifting up and putting down the roller) in relation to maximum neuromuscular activation. To provide accurate results, appropriateness of each electrode's placement was re-checked prior to each repetition.

#### 2.3.2. Subjective Methods

*Revised Oswestry Pain Questionnaire. *The degree of LBP-imposed limitations in ADLs was determined with Revised Oswestry Pain Questionnaire, also referred to as Oswestry Disability Index (ODI). We used this specific modification of the questionnaire, since unlike other instruments, it included a question about changes in lumbar pain intensity. The survey consisted of 10 questions, each with 6 possible responses scored from 0 to 5 points. If the respondent chose more than one answer to a given question, the one with higher score was recorded and subjected to the analysis. Maximum overall score amounted to 50 points, which corresponded to 100% disability due to lumbar pain [18]. Misterska et al. conducted validation and cross-cultural adaptation of Polish version of the Oswestry Questionnaire [18]. Cronbach's alpha, expressing internal consistency rate for the adapted version, was 0.85. The authors of the adaptation emphasized that the questionnaire is an accurate and valid instrument, which makes it a key disability measure in Polish patients with spinal pain [18].

*Roland-Morris Disability Questionnaire (RMDQ). *The second most commonly used questionnaire for the determination of disability associated with lumbar pain is Roland-Morris Disability Questionnaire (RMDQ). The instrument consists of 24 statements about pain and its influence on ADLs. The respondent chooses only the statements that refer to his/her ailments. Maximum overall score may vary between 0 and 24 points. Polish adaptation of RMDQ was prepared by Opara et al. [19]. Cronbach's alpha for the Polish version is 0.88. The results obtained with the Polish version were shown to correlate strongly with the scores determined with other similar instruments (p<0.00001). Polish adaptation of RMDQ proved to be an accurate instrument to determine the degree of LBP-associated disability [19]. RMDQ is not superior to ODI and vice versa, and since these two instruments include different questions, some authors recommend using them together, to fully evaluate the impact of LBP on patient's performance during the ADLs [20].

*Numeric Rating Scale (NRS). *NRS is a most common instrument to assess the severity of a given ailment. It describes subjective intensity of pain on a scale from 0 (no pain) to 10 (worst possible pain). Research showed that NRS and Visual Analog Scale are highly accurate, reliable, and reproducible instruments to determine pain intensity [21]. In our present study, we used NRS to determine the intensity of currently experienced pain and maximum intensity of pain during the last three months.

### 2.4. Statistical Methods

The results (somatic measurements, scores of the scales and surveys used to determine pain intensity and the degree of limitations in the activities of daily living, spondylometric and bioelectrical parameters) were recorded in MS Excel 97-2003 database and subjected to statistical analysis with Statistica package, version 13 (StatSoft).

The methods of mathematical statistics used to analyze the data included Student t-test to verify the significance of differences between two variables and Snedecor's F-test to examine the significance of between-group differences in the values of dependent variables.

To identify the parameters which were influenced most by participation in the general rehabilitation gymnastics program, partial eta-squared values were calculated as an effect measure. The higher the partial eta-squared value, the greater the contribution of the gymnastics to differences in a given parameter between exercising and nonexercising women. A relationship between the bioelectrical activity of the longissimus muscle during various activities and 100% neuromuscular activation of this muscle (analyzed together for the right- and left-sided bundles) was determined on multiple regression analysis.

The results were presented graphically, as the charts and plots of various types.

### 2.5. Ethical Issues

Prior to the study, all women enrolled to the project were informed about its objectives, protocol and methods, and were assured that all the study procedures were noninvasive and safe. Participation in the study was voluntary and free of charge. The participants had the right to withdraw from the study at any time. Moreover, they were assured that before processing, their data will be fully anonymized, and the study results will be used solely for the research purposes, which they consented to. The study included solely the women who gave their written informed consent to participate in the project.

The protocol of the study was approved by the Local Bioethics Committee at the Poznan University of Medical Sciences (decision no. 899/17).

## 3. Results

We compared somatic traits (body height and body weight) and BMI of exercising women and nonexercising controls (see Table 2). Test of the t-Student demonstrated that women who exercised presented with significantly lower BMI than the control (p≤0.01) (see Table 2). This between group difference might correspond to a beneficial effect of general rehabilitation gymnastics. Although minimum and maximum BMI values in both groups were essentially the same, mean BMI for exercising women corresponded to normal body weight, whereas mean BMI for nonexercising controls to lower limit of overweight (according to the international BMI classification for persons older than 65 years) [22].

To analyze the effects of general rehabilitation gymnastics on the ability of the study subjects to undertake ADLs and the intensity of pain caused by chronic LBP, we used three subjective instruments: Revised Oswestry Pain Questionnaire, Roland-Morris Disability Questionnaire, and Numeric Rating Scale (NRS). The results for exercising women and nonexercising controls are presented in Table 3.

Analysis of variance demonstrated significant differences in mean scores for exercising and nonexercising women (see Table 4). Women who participated in the general rehabilitation gymnastics program presented with significantly lower scores for OSW and Rolland-Morris Questionnaire, as well as with significantly lower NRS and NRS max values than the controls. These findings imply that the older women with chronic LBP who undertook general rehabilitation gymnastics experienced fewer limitations in their ADLs and lesser lumbar pain. Statistical analysis demonstrated that general rehabilitation gymnastics exerted the strongest effect on RMDQ scores, contributing to a 40% between group variance in this parameter (as shown by partial eta-squared values; see Table 4).

**Figure 2:**
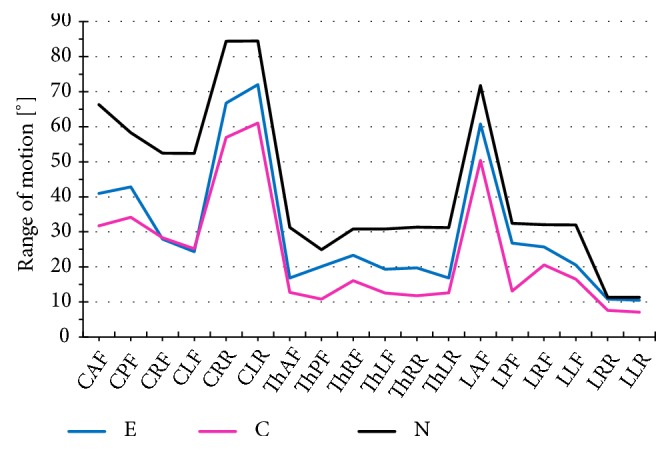
Comparison of functional parameters for various spinal segments in exercising and nonexercising women with normative values according to Lewandowski. (E: exercising group, C: controls, N: normative values for 25-year-old women, CAF: Cervical Anterior Flexion, CPF: Cervical Posterior Flexion, CRF: Cervical Right Flexion, CLF: Cervical Left Flexion, CRR: Cervical Right Rotation, CLR: Cervical Left Rotation, ThAF: Thoracic Anterior Flexion, ThPF: Thoracic Posterior Flexion, ThRF: Thoracic Right Flexion, ThLF - Thoracic Left Flexion, ThRR: Thoracic Right Rotation, ThLR: Thoracic Left Rotation, LAF: Lumbar Anterior Flexion, LPF: Lumbar Posterior Flexion, LRF: Lumbar Right Flexion, LLF: Lumbar Left Flexion, LRR: Lumbar Right Rotation, and LLR: Lumbar Left Rotation).

We also analyzed the effect of general rehabilitation gymnastics (various types of exercises combined with MET) on functional parameters of the spine in women with LBP. To satisfy this objective, we compared spinal mobility in frontal, transverse and sagittal plane in exercising and nonexercising women. Women who participated in the general rehabilitation gymnastics program presented with significantly higher values of all spondylometric parameters. The results are presented on Figure 1, along with mean normative values for 25-year-old women proposed by Lewandowski [15]. Analysis of variance demonstrated significant between group differences in most examined spondylometric parameters (see Table 5). The only parameter that did not differ significantly between the study groups was cervical mobility in frontal plane (CRF and CLF) (see Figure 2).

We used partial eta-squared values to identify the spondylometric parameter that was influenced most by the general rehabilitation gymnastic factor (see Table 5). The higher the value of the eta-squared statistics, the greater the contribution of the gymnastics to between group differences in a given spondylometric parameter. The most evident between group differences were observed in the case of thoracic right rotation (ThRR) and lumbar posterior flexion (LPF); based on partial eta-squared values, general rehabilitation gymnastics contributed to more than 50% between group variance in angular values of these two parameters in exercising and nonexercising women.

Another aim of this study was to analyze the effect of general rehabilitation gymnastics on bioelectrical activity of muscles stabilizing lumbar spine. To satisfy this objective, we compared bioelectrical activity of lumbar portion of the longissimus muscle and its right- and left-sided bundles during various activities undertaken by exercising and nonexercising women.

In both groups, we determined MVC in a prone position with arms crossed under the chin and extended legs, as well as bioelectrical activity during getting up from a chair (Gu), sitting down on a chair (Sd), and lifting up (Lu) and putting down a rehabilitation roller (Pd). Mean activation amplitude during each of these activities was calculated for the whole longissimus muscle and for its right- and left-sided bundles. Although exercising women presented with higher values of all parameters, analysis of variance demonstrated statistically significant between group differences solely for MVC_R_ and MVC_L+R_ (see Table 6). Significantly higher MVC values for the right-sided bundle of the longissimus (MVC_R_) and for the whole muscle (MVC_L+R_) in exercising women imply that general rehabilitation gymnastics exerted a beneficial effect on the strength of muscles stabilizing lumbar spine. Based on partial eta-squared values it can be concluded that general rehabilitation gymnastics contributed to 10% between group variance in MVC_R_ values in exercising and nonexercising women (see Table 6).

Then, we conducted multiple regression analysis to determine a relationship between bioelectrical activity of the longissimus during various activities and its 100% neuromuscular activation (analyzed together for the right- and left-sided bundles). Four independent variables included in the model (Gu_L+R_, Sd_L+R_, Lu_L+R_, and Pd_L+R_) explained 51% of variance in the dependent variable (MVC_L+R_) in exercising women (coefficient of determination = multiple R^2^ = 0.509 x100) and 41% in nonexercising women (coefficient of determination = multiple R^2^ = 0.413 x100) (see Table 7). The results of correlation matrix analysis are presented in Table 8. Some discrepancies between these results and the outcomes of regression analysis stem from the fact that the latter included independent variables that correlated with each other. In exercising women, bioelectrical activity of the longissimus during all activities correlated strongly with its MVC, whereas in nonexercising women, power of the correlations, with the strongest relationships observed for bioelectrical activity during lifting up a rehabilitation roller and the weakest for bioelectrical activity during sitting down on a chair (see Table 8). Probably, the latter finding reflected the way nonexercising women sat on a chair: passively, with only a minimum eccentric contraction of the longissimus.

Subsequently, the amplitudes of the longissimus activation during various activities were divided by the respective MVC values, both for the whole muscle and for its right- and left-sided bundles. As a result, the degree of neuromuscular activation during various activities was expressed as the percentage of MVC (see Table 9). The relative involvement of the longissimus (percentage of MVC) during various activities turned out to be smaller in exercising women than in nonexercising controls, implying that condition of the muscle in the former group was better. However, analysis of variance did not demonstrate statistically significant between group differences in the involvement of the longissimus muscle during various tasks (see Table 10). Analysis of partial eta-squared values identified bioelectrical activity of the right-sided longissimus bundles during standing up from a chair and bioelectrical activity of the whole muscle during lifting up a rehabilitation roller as the parameters having the greatest contribution to between group variance (see Table 10). During both these activities, muscle work must overcome the gravity forces and hence, nonexercising controls whose back muscles were presumably weaker probably required greater activity of the longissimus than exercising women.

## 4. Discussion

### 4.1. Study Limitations

Chronic lower back pain frequently contributes to worse comfort of life in the elderly since strong pain causes serious limitations in the activities of daily living. This makes the topic of this study of utmost importance. A serious limitation of our study was its retrospective character. Nevertheless, the study had also some strengths among which the most important is relatively large size and homogeneity of the study group. The study included older women, aged 65-75 years, who participated in a 3-year general rehabilitation gymnastics program combining strength, stretching, endurance, balance and stabilization exercises with the elements of Muscle Energy Techniques, supervised by the same physiotherapist, a specialist in MET. To the best of our knowledge, the outcome of such complex therapy (general rehabilitation gymnastics + MET) has not been a subject of any previously published study. This highlights the value of our study, conducted in a group of patients who are generally difficult to access. The lack of baseline measurements resulted from the inability to access the exercising women at the time of their enrollment to the gymnastics program. We were able to examine the women no earlier than after three years of a regular exercising. Hence, we decided to compare the results obtained in the exercising group with the results of nonexercising women who presented with the same ailments. Selection of women to both study groups was based on a detailed and thorough analysis. Considering the number of study eligibility criteria, the examined sample can be considered large enough, and in our opinion, the results obtained in such group are sufficient to formulate conclusions important from the practitioner's perspective.

### 4.2. Clinical Practical Implications

#### 4.2.1. Pain Level

Previous studies analyzed effectiveness of various combined exercise programs in attenuation of pain experienced by older persons with chronic LBP. For example, Khalil* et al*. studied the effects of “aggressive physical conditioning” in older persons [23]. Their training program included strength, stretching and balance exercises, and additionally focused on correction of patient's gait and posture. The exercises, performed every day for a period of four weeks, contributed to a 22% decrease in pain severity. Mailloux* et al*. examined the effects of a 6-week spine rehabilitation program for older persons, including a combination of strength, stretching and endurance exercises [24]. In addition to the supervised training sessions held twice a week, the study patients exercised at home. Patients who completed the program declared a 20% decrease in subjective severity of pain measured on Visual Analog Scale. Weiner* et al*. conducted a randomized controlled trial including a group of 200 older persons, to verify the effectiveness of general conditioning and aerobic exercises on a treadmill or cycling ergometer, combined with percutaneous electrical nerve stimulation [25]. Aside from the outcomes of the combined therapy, the authors analyzed also the effects of each of those treatments separately. The program lasted six weeks, with two training sessions per week. When used separately, neither general conditioning nor aerobic exercises contributed to a decrease in pain severity measured with McGill Pain Questionnaire Short-Form, and the level of pain in patients subjected to both therapies was only 9% lower than at the baseline. In another study, conducted by Beissner et al., patients who completed a training program including resistance and stretching exercises, walking and cognitive-behavioral elements, reported a 17% decrease in pain severity expressed on Numeric Rating Scale [26]. According to Hicks et al., 60% of participants of their study experienced a significant reduction of pain (measured with NRS) after participation in a program containing general strength and flexibility exercises, elements of abdominal muscle training, extension of thoracolumbar spine, and scapular retraction [27]. However, 8% of persons participating in this study reported a statistically significant increase in pain severity. The 12-month program included two sessions per week, each lasting one hour [27].

Our study demonstrated that participation in a 3-year training program combining general rehabilitation gymnastics (strength, stretching, conditioning, and stabilization exercises) with elements of MET, divided into 45-min biweekly sessions, contributed to a statistically significant reduction of pain severity (NRS values) in older women (65-75 years of age) with chronic LBP. At the end of the training program, the level of pain in participating women was 62.4% lower than in nonexercising controls, and the between group difference in NRS_max_ values amounted to 31.3%.

Such evident difference in the severity of pain reported by women who engaged in general rehabilitation gymnastics and those who did not, implies that participation in the training might attenuate pain in older persons with chronic LBP. Probably, these were the elements of MET which added considerably to the outcome of the program; the beneficial role of MET in pain attenuation has been already demonstrated by other authors [28, 29].

A statistically significant effect (p<0.05) of nonpharmacological interventions on the severity of chronic pain in older patients was also demonstrated by Park et al. [30]. Reduction of pain severity may be a consequence of beneficial functional and structural changes in lumbar spine. Some exercises induce favorable changes in anulus fibrosus and nucleus pulposus of the intervertebral disc, which may contribute to improvement of its biomechanics [31]. Exercise-based therapies for LBP exert beneficial effect on articular capsules of the intervertebral joints, spinal ligaments, and tendons of muscles located in this area. Moreover, exercise improves blood perfusion of the spinal region, which promotes repair of damaged tissues. One study demonstrated that exercising persons presented with larger cross-section area of spinal extensors and better motor and stabilizing function of the spine than nonexercising controls, which contributed to lesser pain associated with LBP [32].

#### 4.2.2. Disability

Pain associated with LBP imposes limitations on ADLs [33]. A few published studies analyzed changes in disability level after various combined training or rehabilitation programs for older persons with chronic LBP. Mailloux et al. demonstrated that a spine rehabilitation program contributed to a 13% decrease in disability level measured with ODI [24]. Also studies with RMDQ confirmed that some combined exercise programs may reduce disability in older persons with chronic LBP. Beissner et al. reported a 22% decrease in RMDQ scores after combination of physical exercise and cognitive-behavioral elements [26], and Weiner et al. showed an 11% improvement in participants of a program combining physical exercise with percutaneous electrical nerve stimulation [25]. Basler et al. used Hannover Function Ability Questionnaire to verify the effectiveness of a 20-min individual physiotherapy sessions [34]. Each session included a combination of strength, stretching, and endurance exercises. Patients who participated in the rehabilitation program showed an 8% improvement in their disability scores, as compared to a 4% increase in the controls [34].

Women with chronic LBP who participated in our training program presented with lower ODI and RMDQ scores than the controls, by 30% and 65%, respectively; this implies that the exercising women experienced less pain-imposed limitations in ADLs. In our opinion, the substantial difference in the results obtained with the two questionnaires was associated with the fact that they referred to various areas of daily living. This suggests that the degree of disability due to chronic LBP should be assessed with a few various instruments. Irrespective of used questionnaire, the scores for women who participated in the exercise program were significantly different than in the controls, which is consistent with the results reported by other authors. According to Geneen et al., physical activity may contribute to a lesser pain and greater functional improvement not only in the case of LBP [35]. Those authors observed similar beneficial effects of physical exercise in patients with other conditions, such as rheumatoid arthritis, osteoarthritis, fibromyalgia, intermittent claudication, dysmenorrhea, mechanical neck disorder, spinal cord injury, post-polio syndrome, and patellofemoral pain [35].

#### 4.2.3. Spine Mobility

Chronic LBP contributes to a considerable reduction of spine mobility in the affected person. The loss of spinal mobility is likely a consequence of degenerative changes in intervertebral discs [36]. Impaired amortizing function of the disc is reflected by a greater load onto the intervertebral joints. Human spine is a continuous biokinematic chain and therefore, disorders in one intervertebral segment may promote deviations and impairment of mobility in the others [37]. This may lead to hypo- or hypermobility of intervertebral joints, not only in lumbar spine but also in other segments [37]. Coyle et al. compared inclinometer-measured mobility of lumbar spine in older persons with chronic LBP and age-matched healthy controls [38]. Each group included 54 persons aged 60-85 years. The study showed that older persons with chronic LBP had significantly smaller angles of flexion (by 6°, p=0.029) and extension (by nearly 5°, p=0.013) than the controls. The study groups did not differ significantly in terms of the average side-bending. Impaired lumbar mobility in sagittal plane may have detrimental effect on one's performance during ADLs and, hence, should constitute a key parameter during the evaluation and treatment of chronic LBP [38].

In our study, the range of spinal mobility was considerably greater in women who participated in the general rehabilitation gymnastics program than in the controls. The differences for nearly all analyzed parameters were statistically significant and observed regardless of spinal segment. Such substantial difference in the spondylometric parameters might reflect beneficial effect of the general rehabilitation gymnastics on motor function of the spine. The most evident between group difference was observed in the case of lumbar posterior flexion. Perhaps this was associated with the fact that prone lumbar extension was included in each training session and recommended as an exercise to be performed at home. Effectiveness of isolated lumbar extension in patients with chronic LBP was previously documented in a randomized controlled trial conducted by Steele et al. [39]. The only parameter without statistically significant between group differences was cervical lateral flexion. This might be a consequence of degenerative changes within uncovertebral joints. These structures are formed between uncinate processes of a cervical vertebra below and lateral surfaces of the vertebra above [40]. Structural disorders of uncovertebral joints may contribute to lesser mobility of cervical spine during side-bending [41].

To the best of our knowledge, none of the previous studies analyzed the effectiveness of combined exercises on spinal mobility in older persons with chronic LBP. Previous studies centered around the effects of isolated exercises. Helmhout et al. demonstrated that exercises for the erector spinae had beneficial effect on pain and functional disability in persons with chronic LBP, but did not improve lumbar mobility [42]. Bhadauria et al. compared three exercise programs for individuals with chronic LBP, showing that dynamic strengthening, lumbar stabilization and Pilates contribute to a significant improvement in lumbar mobility in sagittal plane [43]. However, it needs to be stressed that the study groups examined by those authors included only 12 persons each, participants varied considerably in terms of age (range 20-60 years), and their spinal mobility was tested solely with modified Schober test [43].

Aside from general rehabilitation gymnastics, our training program included also elements of MET. These therapeutic procedures have been used frequently for the rehabilitation of virtually all body regions, in particular cervical and lumbar spine. Available evidence shows that MET may improve spinal mobility in persons with chronic LBP. Unfortunately, many of the studies dealing with the problem in question had some methodological flaws [44].

#### 4.2.4. Electromyographic Parameters

Chronic LBP is postulated to play a significant role in the erector spinae dysfunction, which manifests as lower values of mean amplitude, mean density, and maximum amplitude for this muscle group in lumbar and thoracic region. Chronic LBP exerts the most detrimental effect on type I muscle fibers [45]. A dysfunction of the erector spinae may result in spinal overload during dynamic and static work [46].

To the best of our knowledge, none of the published studies analyzed the effects of combined exercises in patients with chronic LBP in the context of changes in bioelectrical activity of back muscles. Few papers dealing with the problem in question examined the outcomes of isolated interventions. A statistically significant (P < 0.05) change in muscle activity was observed in women with postpartum LBP, subjected to posteroanterior lumbar mobilization. Significantly lower surface EMG activity of the erector spinae might correspond to lesser ailments in this region [47]. Also Postural Taping with nonelastic tape was shown to exert a beneficial effect in patients with chronic LBP. The protective effect of the therapy manifested as a significant (p<0.001) decrease in bioelectrical muscle activity during dynamic tasks [48]. However, no statistically significant difference in surface EMG activity was observed in patients with chronic LBP subjected to Traditional Bone Setting and Conventional Physical Therapy [49]. Also electroacupuncture exerted no significant effect on EMG activity [50].

In our study, we did not observe a significant difference in the bioelectrical activity of the longissimus dorsi muscle in women participating in general rehabilitation gymnastics program with elements of MET and in nonexercising controls. Perhaps, the lack of statistically significant between group differences might be a consequence of less demanding tasks used during EMG testing (standing up/seating down and lifting up/putting down a rehabilitation roller).

### 4.3. Future Research

Future studies should comprehensively evaluate the effectiveness of the complex therapy (general rehabilitation gymnastics + MET). Since CLBP is a common ailment, future research could be expanded onto various age groups of patients. Optimally, the problem in question should be a subject of a randomized clinical trial. An additional advantage of such study would be the possibility of a follow-up evaluation, to verify whether the beneficial effects of the therapy might persist longer.

## 5. Conclusions

The following conclusions can be formulated based on the results of this study:

1. Older women with chronic LBP who participated in a general rehabilitation gymnastic program with the elements of muscle energy techniques (MET) presented with significantly lower pain, lesser limitations in the activities of daily living, and better spinal mobility. It can be assumed that the difference in the subjective and objective parameters of exercising and nonexercising women was a consequence of participation in the gymnastics program.
2. The study groups did not differ significantly in terms of the amplitudes of bioelectrical signal from the back muscles, which might result from the use of a less burdensome EMG test (standing up from a chair, sitting down on a chair, and lifting up and putting down a 2-kg rehabilitation roller).

## Figures and Tables

**Table 1:** Demographic and clinical characteristics of the study participants.

Parameter	Category	Exercising group		Control group	
		N	%	N	%
Age	65-70 years	13	62	15	75
	71-75 years	8	38	5	25

Education	Higher	7	33.3	6	30
	Secondary	14	66.7	14	70

Type of occupational activity	Sedentary 8 hours	10	47.6	5	25
	Sedentary 4 out of 8 hours	6	28.6	7	35
	Physical	4	19	6	30
	No occupational activity	1	4.8	2	10

Place of residence	Town	15	71.4	12	60
	County	6	28.6	8	40

Economic status	High	6	28.6	7	35
	Moderate	15	71.4	13	65

Location of pain	Central pain in the spine	5	23.8	7	35
	Left-sided	6	28.6	3	15
	Right-sided	7	33.3	6	30
	Spine + buttock	3	14.3	4	20

**Table 2:** Characteristics of the study participants. Comparison of the studied groups by the Student's t-test.

Parameter	Exercising group				Non-exercising controls				
	N = 21				N = 20				
	Mean	SD	MIN	MAX	Mean	SD	MIN	MAX	p

Body height [cm]	161.1	6.7	148	173	157.2	6.9	144	170	0.067
Body weight [kg]	72.2	10.4	56	91	74.4	10.4	55	90	0.510
BMI [kg/m^2^]	27.5	3.0	21.9	34.7	30.0	3.0	21.8	34.1	0.010*∗∗*
Age [years]	69.1	3.9	65	75	67.8	3.3	65	75	0.240

N: number, SD: standard deviation, p: probability of a type I error, *∗*: statistically significant differences at *α*≤ 0.05, and *∗∗*: statistically significant differences at *α*≤0.01

**Table 3:** Statistical characteristics of questionnaire (OSW, Rolland-Morris, NRS and NRS max) scores for exercising women and non-exercising controls.

Group		ODI		RMDQ		NRS		NRS max	
	N	Mean	SD	Mean	SD	Mean	SD	Mean	SD
Exercising	21	10.62	5.46	2.48	2.14	1.43	1.69	4.95	2.25
Controls	20	15.15	5.61	7.10	3.49	3.80	2.07	7.20	0.89

N: number, SD: standard deviation, ODI: Oswestry Disability Index, RMDQ: Roland-Morris Disability Questionnaire, and NRS: Numeric Rating Scale.

**Table 4:** Effect of general rehabilitation gymnastics on selected dependent variables (questionnaire scores); analysis of variance.

Variable	df_1_	df_2_	F	p	Partial eta-squared	%
ODI	1	39	6.86	0.0125*∗*	0.1496	14.960
RMDQ	1	39	26.44	0.0001*∗∗*	0.4040	40.404
NRS	1	39	16.24	0.0003*∗∗*	0.2940	29.399
NRS max	1	39	17.38	0.0002*∗∗*	0.3082	30.822

ODI: Oswestry Disability Index, RMDQ: Roland-Morris Disability Questionnaire, NRS: Numeric Rating Scale, df: degrees of freedom, F: value of the Snedecor F-test, p: probability of a type I error, *∗*: statistically significant differences at *α*≤ 0.05, and *∗∗*: statistically significant differences at *α*≤0.01.

**Table 5:** Effect of general rehabilitation gymnastics on spondylometric parameters of the spine in women with chronic low back pain, analysis of variance.

Variable	df_1_	df_2_	F	p	Partial eta-squared	%
CAF	1	39	13.55	0.001*∗∗*	0.26	25.79
CPF	1	39	10.82	0.001*∗∗*	0.22	21.72
CRF	1	39	0.03	0.87	0.00	0.07
CLF	1	39	0.17	0.69	0.00	0.42
CRR	1	39	7.16	0.01*∗∗*	0.16	15.52
CLR	1	39	12.29	0.001*∗∗*	0.24	23.96
ThAF	1	39	4.24	0.05*∗*	0.10	9.80
ThPF	1	39	18.17	0.001*∗∗*	0.32	31.78
ThRF	1	39	17.18	0.001*∗∗*	0.31	30.58
ThLF	1	39	27.62	0.001*∗∗*	0.41	41.46
ThRR	1	39	46.13	0.001*∗∗*	0.54	54.19
ThLR	1	39	6.39	0.02*∗*	0.14	14.08
LAF	1	39	6.24	0.02*∗*	0.14	13.79
LPF	1	39	40.05	0.001*∗∗*	0.51	50.66
LRF	1	39	8.32	0.01*∗∗*	0.18	17.58
LLF	1	39	7.34	0.01*∗∗*	0.16	15.84
LRR	1	39	5.56	0.02*∗*	0.12	12.47
LLR	1	39	6.06	0.02*∗*	0.13	13.46

CAF: Cervical Anterior Flexion, CPF: Cervical Posterior Flexion, CRF: Cervical Right Flexion, CLF: Cervical Left Flexion, CRR: Cervical Right Rotation, CLR: Cervical Left Rotation, ThAF: Thoracic Anterior Flexion, ThPF: Thoracic Posterior Flexion, ThRF: Thoracic Right Flexion, ThLF - Thoracic Left Flexion, ThRR: Thoracic Right Rotation, ThLR: Thoracic Left Rotation, LAF: Lumbar Anterior Flexion, LPF: Lumbar Posterior Flexion, LRF: Lumbar Right Flexion, LLF: Lumbar Left Flexion, LRR: Lumbar Right Rotation, LLR: Lumbar Left Rotation, df: degrees of freedom, F: value of the Snedecor F-test, p: probability of a type I error, *∗*: statistically significant differences at *α*≤ 0.05, and *∗∗*: statistically significant differences at *α*≤0.01.

**Table 6:** Effect of general rehabilitation gymnastics on neuromuscular activation of the longissimus muscle during various activities undertaken by women with chronic LBP, analysis of variance.

Variable	df_1_	df_2_	F	p	Partial eta-squared	%
MVC-Mean_L_	1	39	2.84	0.0999	0.0679	6.785
MVC-Mean_R_	1	39	4.57	0.0389*∗*	0.1048	10.481
MVC-Mean_L+R_	1	39	3.78	0.0500*∗*	0.0884	8.844
Pd-Mean_L_	1	39	1.31	0.2587	0.0326	3.258
Pd-Mean_R_	1	39	0.32	0.5747	0.0081	0.815
Pd-Mean_L+R_	1	39	0.92	0.3433	0.0231	2.306
Lu-Mean_L_	1	39	0.31	0.5825	0.0078	0.782
Lu-Mean_R_	1	39	0.05	0.8222	0.0013	0.131
Lu-Mean_L+R_	1	39	0.19	0.6651	0.0049	0.485
Sd-Mean_L_	1	39	0.93	0.3414	0.0232	2.323
Sd-Mean_R_	1	39	0.36	0.5542	0.0090	0.904
Sd-Mean_L+R_	1	39	0.00	0.9850	9.1960	0.001
Gu-Mean_L_	1	39	1.17	0.2864	0.0291	2.909
Gu-Mean_R_	1	39	0.13	0.7160	0.0034	0.343
Gu-Mean_L+R_	1	39	0.69	0.4121	0.0173	1.732

MVC: maximum bioelectrical activity of the longissimus muscle, MVC_R_: maximum bioelectrical activity of the longissimus muscle right-sided, MVC_L_: maximum bioelectrical activity of the longissimus muscle left-sided, MVC_L+R_: maximum bioelectrical activity of the longissimus muscle left- and right sided, Pd: bioelectrical activity of the longissimus muscle during putting down a rehabilitation roller, Lu: bioelectrical activity of the longissimus muscle during lifting up a rehabilitation roller, Sd: bioelectrical activity of the longissimus muscle during sitting down on a chair, Gu: bioelectrical activity of the longissimus muscle during getting up from a chair, df: degrees of freedom, F: value of the Snedecor F-test, p: probability of a type I error, and *∗*: statistically significant differences at *α*≤ 0.05

**Table 7:** Results of multiple regression analysis for MVC_L+P_ as a dependent variable and four independent variables (Gu_L+R_, Sd_L+R_, Lu_L+R_, Pd_L+R_).

Independent variables	Exercising group, R^2^=0.509		Non-exercising controls, R^2^=0.413	
	b*∗*	p	b*∗*	p
Pd_L+P_	0.311	0.378	0.035	0.923
Lu_L+P_	0.335	0.203	0.465	0.242
Sd_L+P_	0.212	0.368	0.226	0.287
Gu_L+P_	0.001	0.999	0.085	0.764

R^2^: coefficient of determination, b*∗*: standardized regression coefficient, p: probability of a type I error.

Pd_L+P_: bioelectrical activity of the longissimus muscle on both sides during putting down a rehabilitation roller.

Lu_L+P_: bioelectrical activity of the longissimus muscle on both sides during lifting up a rehabilitation roller.

Sd_L+P_: bioelectrical activity of the longissimus muscle on both sides during sitting down on a chair.

Gu_L+P_: bioelectrical activity of the longissimus muscle on both sides during getting up from a chair.

**Table 8:** Correlation matrix illustrating power of relationships between the dependent variable MVC_L+P_ and independent variables (Pd_L+P_, Lu_L+P_, Sd_L+P_, Gu_L+P_), coefficients of correlation for the study groups.

Independent variables	Exercising group - MVC_L+P_		Non-exercising controls - MVC_L+P_	
	Correlation coefficient	p	Correlation coefficient	p
Pd_L+P_	0.611	0.003	0.523	0.018
Lu_L+P_	0.595	0.004	0.596	0.006
Sd_L+P_	0.565	0.008	0.342	0.140
Gu_L+P_	0.623	0.003	0.480	0.033

p: probability of a type I error.

MVC_L+R_: maximum bioelectrical activity of the longissimus muscle left- and right sided.

Pd_L+P_: bioelectrical activity of the longissimus muscle on both sides during putting down a rehabilitation roller.

Lu_L+P_: bioelectrical activity of the longissimus muscle on both sides during lifting up a rehabilitation roller.

Sd_L+P_: bioelectrical activity of the longissimus muscle on both sides during sitting down on a chair.

Gu_L+P_: bioelectrical activity of the longissimus muscle on both sides during getting up from a chair.

**Table 9:** Mean values of bioelectrical activity to maximum voluntary contraction (MVC) ratios for the longissimus muscle in exercising women (E) and nonexercising controls (C) during various tasks.

Ratio	Pd_L_/MVC	Pd_P_/MVC	Pd_L+P_/MVC	Lu_L_/MVC	Lu_P_/MVC	Lu_L+P_/MVC
E	51.59	49.55	50.4	48.12	46.54	47.18
C	58.59	62.2	60.14	57.24	59.4	57.93

Ratio	Sd_L_/MVC	Sd_P_/MVC	Sd_L+P_/MVC	Gu_L_/MVC	Gu_P_/MVC	Gu_L+P_/MVC

E	40.96	32.64	36.62	59.24	50.93	54.9
C	45.71	48.00	46.63	69.57	64.45	65.97

E: exercising group; C: controls; MVC: maximum bioelectrical activity of the longissimus muscle; Pd: bioelectrical activity of the longissimus muscle during putting down a rehabilitation roller; Lu: bioelectrical activity of the longissimus muscle during lifting up a rehabilitation roller; Sd: bioelectrical activity of the longissimus muscle during sitting down on a chair; Gu: bioelectrical activity of the longissimus muscle during getting up from a chair.

**Table 10:** Effect of general rehabilitation gymnastics on the relative involvement of the longissimus muscle (expressed as the percentage of its maximum voluntary contraction) during selected activities in women with chronic low back pain.

Variable	df_1_	df_2_	F	p	Partial eta-squared	%
Pd_L_/MVC	1	39	1.27	0.2667	0.0315	3.152
Pd_P_/MVC	1	39	2.32	0.1355	0.0562	5.621
Pd_L+P_/MVC	1	39	2.50	0.1221	0.0602	6.019
Lu_L_/MVC	1	39	2.64	0.1120	0.0635	6.348
Lu_P_/MVC	1	39	3.10	0.0859	0.0737	7.373
Lu_L+P_/MVC	1	39	3.48	0.0698	0.0818	8.182
Sd_L_/MVC	1	39	0.50	0.4849	0.0126	1.259
Sd_P_/MVC	1	39	2.04	0.1610	0.0497	4.975
Sd_L+P_/MVC	1	39	2.51	0.1213	0.0604	6.044
Gu_L_/MVC	1	39	0.91	0.3455	0.0228	2.285
Gu_P_/MVC	1	39	3.73	0.0606	0.0874	8.739
Gu_L+P_/MVC	1	39	2.18	0.1481	0.0529	5.288

MVC: maximum bioelectrical activity of the longissimus muscle; Pd: bioelectrical activity of the longissimus muscle during putting down a rehabilitation roller; Lu: bioelectrical activity of the longissimus muscle during lifting up a rehabilitation roller; Sd: bioelectrical activity of the longissimus muscle during sitting down on a chair; Gu: bioelectrical activity of the longissimus muscle during getting up from a chair; df: degrees of freedom, F: value of the Snedecor F-test, p: probability of a type I error;*∗*: statistically significant differences at *α*≤ 0.05, and *∗∗*: statistically significant differences at *α*≤0.01.

## Data Availability

The data used to support the findings of this study are included within the article. The raw data collected during the study are available from the corresponding author upon request.
